# A method for quantifying vertical stratification in crude oil emulsions by rotational viscometry

**DOI:** 10.1016/j.mex.2026.103848

**Published:** 2026-02-27

**Authors:** Máté Hartyányi, Roland Nagy

**Affiliations:** University of Pannonia, Faculty of Engineering, Department of MOL Hydrocarbon and Coal Processing, Egyetem u. 10., Veszprém H-8200, Hungary

**Keywords:** Stratification index, Vertical heterogeneity, Apparent viscosity, Brookfield viscometer, Emulsion stability, Crude oil emulsions

## Abstract

Vertical stratification during quiescent storage can strongly affect the stability and handling of crude oil–produced water–surfactant emulsions, yet routine methods rarely provide quantitative, height-resolved information on the evolving mechanical state. This paper describes a practical workflow to characterize stratification and aging using a Brookfield-type rotational viscometer operated under fixed spindle–speed–temperature conditions. Apparent viscosity is measured at predefined relative height positions along a liquid column and repeated at scheduled aging times, yielding a spatiotemporal apparent viscosity map. Vertical heterogeneity is quantified by a dimensionless Stratification Index, SI(t), defined as the mean absolute deviation of the height-dependent viscosity profile from the column-averaged viscosity, normalized by the column average at each time point. The resulting SI(t) time series enables direct, comparable assessment of stratification magnitude and evolution across formulations with different absolute viscosity levels. Optional kinetic descriptors, including stratification onset time determined via an objective threshold and characteristic stratification rates from simple saturation models, can be extracted to support comparative screening. Method validation is addressed through technical repeatability, batch-to-batch reproducibility, and null testing with homogeneous reference fluids, with optional comparison to independent stratification indicators.

## Highlights

• Height-resolved viscometry quantifies vertical stratification.
• Apparent viscosity profiles are reduced to a dimensionless SI.
• Enables reproducible comparison of emulsion aging behavior.

## Graphical abstract

**Figure:**
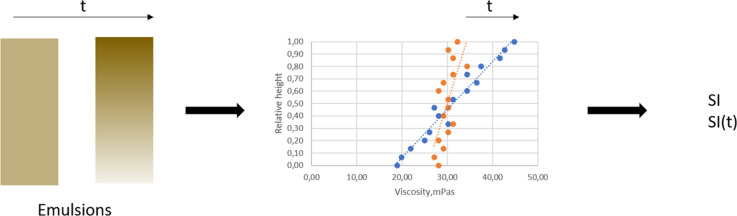

**Table:** Specifications table.

**Subject area**	Chemical Engineering
**More specific subject area**	Oil recovery
**Name of your method**	Height-Resolved Apparent Viscosity Profiling (HR-AVP)
**Name and reference of original method**	Not applicable
**Resource availability**	Not applicable

## Background

Emulsions encountered in petroleum-related operations—such as crude oil–produced water systems stabilized or modified by surfactants—rarely remain perfectly homogeneous during storage or quiescent aging. Even when initially well dispersed, gravitational separation (creaming or sedimentation) and microstructural evolution (flocculation and coalescence) frequently produce vertical gradients in the dispersed-phase volume fraction and droplet interactions, leading to spatially varying mechanical properties. Consequently, viscosity in aging emulsions is often not a single bulk quantity but a spatiotemporal state variable more appropriately described as η(z,t) [mPa s] rather than as η(t) [mPa s] [[1], [2], [3], [4]]. This is practically relevant because viscosity governs pumpability, pressure drop, separation efficiency, and the onset of operational challenges such as poor dehydration or unstable flow behavior [5,6].

Rheology is widely recognized as a structure-sensitive indicator of dispersions and emulsions: emulsion viscosity strongly depends on the dispersed-phase volume fraction, droplet size distribution, droplet deformability, and interdroplet interactions [1,7,8]. In concentrated or weakly attractive systems, small changes in the microstructure can cause large viscosity changes, whereas flocculated networks may introduce pronounced non-Newtonian and time-dependent behavior [[7], [8], [9]]. These features make viscosity a valuable proxy for microstructural stability, especially under low-shear conditions that approximate storage environments (“shelf viscosity” concept) [9,10]. However, most rheology-based stability assessments rely on single-point measurements or homogenized samples, which can be misleading when stratification develops: a viscosity value measured near the top of a column may represent a droplet-enriched cream layer, whereas a measurement near the bottom may represent a depleted zone, yielding contradictory interpretations from the same sample [1,11].

Brookfield-type rotational viscometers provide a pragmatic and broadly accessible route to apparent viscosity measurement in industrial and laboratory settings. Although the measured value is operational (dependent on spindle geometry, rotation speed, and shear history), rotational viscometry is an accepted approach for the comparative characterization of non-Newtonian and time-dependent materials when conditions are standardized [[12], [13], [14]]. Prior petroleum-focused studies have demonstrated the applicability of the Brookfield method to crude oil–in–water emulsions, including measurements in settled states, supporting its feasibility for practical emulsion monitoring [15].

To address the limitations of bulk viscosity in stratifying systems, the present method extends rotational viscometry to height-resolved apparent viscosity profiling, constructing η(z,t) [mPa s] maps over a defined vertical grid and aging timeline. The profiles are converted into a dimensionless stratification index (SI) [-] that quantifies deviations from the column-averaged viscosity, enabling robust comparison across formulations despite differences in absolute viscosity. Tracking SI(t) [-] provides a compact descriptor of stratification kinetics and aging, facilitating formulation screening and validation against independent stability measures (e.g., optical layer evolution or separated-phase volume) [[1], [2], [3],^11^].

## Method details

The aim of this method is to quantitatively characterize the vertical stratification and time-dependent aging of crude oil–produced water–surfactant emulsions during storage using height-resolved apparent viscosity measurements performed with a Brookfield rotational viscometer. The procedure yields a spatiotemporal map of apparent viscosity, η(z,t) [mPa s], which is subsequently transformed into a dimensionless stratification index (SI) and its time-dependent profile, SI(t) [-]. Optionally, kinetic descriptors (e.g., stratification onset time and stratification rate) can be derived from the SI(t) curve, enabling robust and comparable assessment of stratification dynamics across different emulsion formulations.

In this method, the value obtained from the Brookfield rotational viscometer is interpreted as an apparent viscosity, which serves as a reproducible operational indicator of the local mechanical state of the emulsion under a defined spindle–rotation speed–temperature–geometry configuration. Within the sample column, the measurement position is specified by the height z [cm] measured from the bottom, and a normalized relative coordinate z/H is used to enable comparability across different column geometries. By repeating the measurements at multiple relative heights and at multiple time points, a spatiotemporal map of apparent viscosity, η(z,t) [mPa s], is constructed. On the basis of this map, the degree of vertical nonuniformity is quantified using a dimensionless stratification index (SI) [-], which at a given time point expresses the deviation of the height-dependent viscosity profile from the column-averaged viscosity.

The measurement outputs obtained using the Brookfield rotational viscometer are interpreted in the present method as apparent viscosity, which serves as a reproducible operational indicator of the local mechanical state of the emulsion under a defined spindle–rotation speed–temperature–geometry configuration. Within the sample column, the measurement position is specified by the height z measured from the bottom, and a relative height is defined by normalizing z with the total liquid column height H (Eq. (1)):

(1) $$\begin{array}{ccc} z^{*}=\frac{z}{H}; & z\in \left[0,\;H\right]; & z^{*}\in \left[0,\;1\right] \end{array}$$

where *z* = 0 [cm]corresponds to the bottom of the column and *z* = *H* [cm]corresponds to the top. Measurements are performed at N [pcs] fixed relative heights z*_i_ (*i* = 1,…,N) and at multiple time points t, thereby yielding a spatiotemporal map of apparent viscosity, η(z*,t) [mPa s]. At a given time point, the column-averaged apparent viscosity is calculated as the mean of the values obtained at the selected height positions (Eq. (2)):

(2) $$\bar{\eta }\left(t\right)=\frac{1}{N}\sum_{i=1}^{N}\eta \left(z_{i}^{*},t\right)$$

As a dimensionless measure of vertical nonuniformity, the stratification index (SI) [-] is defined as the ratio of the mean absolute deviation of the height-resolved viscosity values from the column-averaged viscosity to the column-averaged viscosity itself (Eq. (3)):

(3) $$SI\left(t\right)=\frac{1}{N\bar{\eta }\left(t\right)}\sum_{i=1}^{N}\left|\eta \left(z_{i}^{*},t\right)-\bar{\eta }\left(t\right)\right|$$

According to the above definition, SI(t)≥0 [-]for all time points t, and SI(t)=0 [-]holds when the viscosity is homogeneous across the measured height positions at that time point, i.e., $\bar{\eta }\left(z_{i}^{*},t\right)=\bar{\eta }\left(t\right)$ [mPa s]for all i. The dimensionless normalization of the SI [-] ensures that stratification can be compared across emulsions with different absolute viscosity levels, while the SI [-] quantitatively captures the mechanical dispersion of the height-dependent viscosity profile.

The method is designed for the investigation of crude oil–produced water–surfactant emulsions, which consist of an oil phase, an aqueous phase with a defined salinity (produced water or synthetic brine), and a selected surface-active agent (surfactant). The emulsion composition is characterized by the oil volume fraction, ϕ_oil_, the composition of the aqueous phase (e.g., total dissolved solids), and the surfactant concentration, c_surf_. The method is applicable to both oil-in-water (O/W) and water-in-oil (W/O) emulsions. During the experiments, the onset of the aging timescale (*t* = 0) is linked to a clearly defined point in the sample preparation workflow (typically after completion of emulsification and transfer of the sample into the measurement column). The emulsion is then stored under quiescent conditions at a controlled temperature, and height-resolved viscosity profiles are recorded at predefined time points.

Height-resolved viscosity profiles are recorded at predefined time points according to a fixed measurement schedule to enable comparable tracking of the temporal evolution of aging and stratification. When the measurement times t_j_(*j* = 1,…,M) [min] are selected, the sampling density is adjusted to the expected dynamics of the emulsion: denser sampling is recommended during the early period, when rapid structural rearrangement or gravitational separation may occur, whereas less frequent time points may be sufficient at later stages to capture slower changes. Accordingly, the measurement schedule is typically designed to cover two regimes: (i) a short, high-resolution early phase that ensures detection of the onset of stratification and (ii) a longer, lower-frequency late phase suitable for documenting stabilization trends or progression toward phase separation. As a result of repeating viscosity profiling over time, an *N* × *M* data matrix η(z*,t_j_) [mPa s] is obtained, which forms the basis for the subsequent computation of SI(t) [-] and kinetic interpretation.

Apparent viscosity measurements are performed using a Brookfield-type rotational viscometer (instrument model: Brookfield Amatek DVNext) equipped with a spindle appropriate for the investigated viscosity range (spindle type: helipath spindle). During measurement, the rotation speed is fixed, and the temperature is maintained constant throughout the entire measurement series (T_meas_=80 °C) using an appropriate temperature-controlled setup (jacketed vessel). Instrument setup and verification are performed according to the manufacturer’s recommendations, and the torque signal is kept within the reliable operating range; the combination of the spindle–rotation speed is selected to ensure that the measurement signal remains stable and reproducible across the full viscosity range of the tested samples. To ensure reproducibility, each experiment should report the instrument model, spindle identifier, rotation speed (or the list of rotation speeds used), measurement temperature, and temperature-control method.

From the height-resolved spatiotemporal viscosity map η(z*,t_j_) [mPa s], the temporal evolution of vertical nonuniformity is summarized using a single dimensionless descriptor defined as the stratification index (SI) [-]. The calculation of SI(t) [-] is performed for each measurement time point t_j_ [min] on the basis of the definition provided above; i.e., the mean absolute deviation of the height-dependent viscosity values from the column-averaged viscosity is normalized by the corresponding column-averaged viscosity at that time. This results in an M-point SI(t) time series, which enables a comparable quantification of the magnitude and dynamics of stratification across different emulsion compositions and experimental conditions. To assess method robustness, alternative forms of the SI [-] can also be computed (e.g., the ratio of the standard deviation of the viscosity profile to its mean or the viscosity ratio between the top and bottom measurement positions), which can be used to evaluate the sensitivity of stratification-related conclusions to the chosen index definition.

## Method validation

During method validation, we explicitly define the validated outputs as the height-resolved spatiotemporal map of apparent viscosity, η(z*,t) [mPa s], obtained with a Brookfield rotational viscometer, the derived stratification index time series, SI(t), and—where applicable—the associated kinetic descriptors (e.g., the stratification onset time t_sep_, the characteristic rate constant k, and the asymptotic stratification level SI_∞_). The purpose of validation is not to establish intrinsic rheological material properties but to demonstrate that the proposed workflow yields reproducible and interpretable stratification metrics under standardized measurement conditions, thereby enabling robust, quantitative comparisons of stratification behavior across different crude oil–produced water–surfactant emulsion formulations and experimental settings.

To assess the technical repeatability of the height-resolved apparent viscosity measurements, a representative emulsion sample was analysed at a fixed aging time ([*t* = 0 min]) under identical instrumental and environmental conditions. Apparent viscosity measurements were performed at [*N* = 3] predefined relative height positions (z_i_*) along the sample column, and at each height position [*n* = 5], technical replicates were recorded without altering the measurement settings. For each z_i_*, the mean apparent viscosity, standard deviation, and relative standard deviation were calculated from the replicate measurements and are shown in Table 1.

**Table 1:** Validation of the technical repeatability of viscosity measurements.

z*	mean η [mPa s]	standard deviation [mPas]	RSD %
1.00	32.59	0.36	1.12
0.53	30.99	0.80	2.58
0.00	28.28	0.14	0.48

These statistics provide a quantitative estimate of the measurement noise associated with the Brookfield-based profiling procedure and define the uncertainty level that propagates into the derived spatiotemporal viscosity map η(z*,t) and the subsequently calculated stratification index.

To quantify the technical repeatability of the stratification index (SI), a representative emulsion sample was analysed at a fixed aging time [*t* = 0 and 60 min] under identical measurement conditions. The height-resolved apparent viscosity profile was acquired with [*n* = 3] technical replicates using the same instrumental settings. For each replicate, the SI was computed from the corresponding set of η(z_i_*,t) values measured at [*N* = 16] predefined relative height positions (z_i_*) according to the definition introduced above. The resulting distribution of [*n* = 3] SI values was summarized by the mean, standard deviation, and relative standard deviation, providing a quantitative estimate of how instrumental and procedural variability in the viscosity profiling step propagates into the derived, dimensionless stratification metric. These results are summarized in Table 2.

**Table 2:** Validation of the technical repeatability of the stratification index.

t	mean SI [-]	standard deviation	RSD %
0 min	0.048	0.002	3.91
60 min	0.212	0.001	0.28

Batch-to-batch reproducibility was evaluated using [*m* = 3] independently prepared emulsions of identical formulations produced under the same preparation and storage conditions. Height-resolved apparent viscosity profiles were recorded for each batch at the same predefined relative height grid [*N* = 16] and at fixed time points [*t* = 0 and 60 min], after which the SI(t) values were computed for each time point. Reproducibility was quantified at each time point by the between-batch mean, standard deviation and RSD % of the SI, thereby capturing variability introduced by independent sample preparation in addition to measurement variability. The results are shown in Table 3.

**Table 3:** Validation of the Batch-to-Batch Reproducibility of the Stratification Index.

t	mean SI	standard deviation	RSD %
0 min	0.047	0.002	4.10
60 min	0.210	0.002	0.72

A homogeneous reference null test was performed to rule out geometrical or instrument-induced artifacts in the SI calculation. A nonstratifying, homogeneous reference fluid (brine) was loaded into the measurement column, and the apparent viscosity was measured across the full relative height grid [*N* = 16] positions using the same instrument configuration as applied to the emulsions. Measurements were repeated [*n* = 3] times at each height position, and the SI was computed from the resulting viscosity profile. The null test was considered successful because the η(z*) profile was invariant within measurement uncertainty and the computed SI values remained close to zero (SI_null_=0002) within the noise level, confirming that the nonzero SI responses observed for emulsions arise from genuine vertical heterogeneity rather than measurement or geometry artifacts.

## Limitations

The proposed workflow is based on the operational apparent viscosity obtained by Brookfield-type rotational viscometry; therefore, the reported values and derived stratification descriptors are specific to the selected spindle–rotation speed–temperature–geometry configuration and should not be interpreted as intrinsic rheological material properties. As a consequence, comparisons across studies require consistent instrument settings, and within-study comparisons should be performed under a fixed measurement protocol. This method is best suited to systems whose stratification and aging timescales are comparable to or longer than the time required to acquire a full height profile; if stratification evolves rapidly during profiling, the resulting η(z*,t) map may reflect a convolution of spatial variation and time evolution within a single measurement cycle. Because measurements impose shear locally and the spindle must be repositioned between heights, fragile emulsions may be partially perturbed, potentially attenuating the true degree of vertical heterogeneity; this risk increases for systems prone to rapid coalescence, strong thixotropy, or gel-like structure formation. Practical constraints also arise from the torque operating window of the instrument: samples that are too low in viscosity may yield unstable signals, whereas highly viscous or highly heterogeneous regions may exceed the reliable torque range, limiting the accessible height domain or necessitating changes in the spindle or speed. Finally, when aging leads to near-complete phase separation (e.g., distinct oil- and water-rich layers), some height positions may no longer represent an emulsion microstructure; in such cases, the SI remains a useful descriptor of vertical nonuniformity, but interpretation should explicitly acknowledge that the profile includes measurements of different phases rather than a single emulsion continuum.

Because Brookfield measurements provide apparent viscosity under defined spindle–speed conditions rather than a controlled shear-rate rheological parameter, changes in rotational speed may alter absolute viscosity values; therefore, SI-based comparisons should be performed under fixed instrument settings.

## Ethics statements

The authors confirm that this work complies with the ethical requirements for publication in MethodsX. The study does not involve human participants, human data, or animal experiments.

## CRediT author statement

Máté Hartyányi: Writing, Conceptualization, Investigation, Formal Analysis, Methodology

Roland Nagy: Supervision

## Declaration of generative AI and AI-assisted technologies in the manuscript preparation process

During the preparation of this work, the author(s) used ChatGPT for language editing and structuring of the manuscript text and Rubriq for language check. After using this tool/service, the author(s) reviewed and edited the content as needed and take(s) full responsibility for the content of the published article.

## Declaration of competing interest

The authors declare that they have no known competing financial interests or personal relationships that could have appeared to influence the work reported in this paper.

## Data Availability

Data will be made available on request.
